# Multistructural and Multiscale Instability Characterization of Gas–Liquid Two-Phase Flow with MRA-CMESSE

**DOI:** 10.3390/e28040403

**Published:** 2026-04-02

**Authors:** Qing-Ming Sun, Qing-Chao Yu, Di Ba

**Affiliations:** 1 School of Mechanical and Electrical, Qiqihar University, Qiqihar 161006, China; 2 The Engineering Technology Research Center for Precision Manufacturing Equipment and Industrial Perception of Heilongjiang Province, Qiqihar 161006, China; 3 The Collaborative Innovation Center for Intelligent Manufacturing Equipment Industrialization, Qiqihar 161006, China

**Keywords:** multiscale symbolic entropy, wavelet multiresolution analysis, pressure fluctuation analysis, vertical gas–liquid flow, instability quantification

## Abstract

Characterizing instability in gas–liquid flows is difficult because flow dynamics interact across multiple scales. In this work, we develop an integrated framework that combines multi-resolution analysis with composite multiscale equiprobable symbolic sample entropy (MRA-CMESSE). This combination enables us to examine flow instability from a multistructural and multiscale perspective. A comprehensive evaluation across four distinct metrics shows that our method is more robust to changes in data length than multiscale sample entropy and composite multiscale sample entropy approaches. Furthermore, MRA-CMESSE is applied to analyze differential pressure time series from vertical air–water two-phase flow, providing a quantitative characterization of the instability of three flow patterns. Among these, bubble flow is the most unstable, with energy spread out and high complexity at small scales; slug flow is the most stable, with its energy focused at larger scales with low complexity, and churn flow falls in between. A central finding is that as superficial gas velocity increases, energy and complexity shift to the meso-scale and micro-scale. This quantitative analysis identifies increased agitation at the meso-scale and micro-scale as the primary driver of enhanced overall flow instability. This framework offers a new quantitative basis for analyzing gas–liquid two-phase flows and strengthens the physical foundation for the monitoring and control of related industrial systems.

## 1. Introduction

Flow instability in gas–liquid two-phase flow strongly affects the safety and operational efficiency of key industrial applications, including petroleum refining, chemical engineering, and power generation [1,2,3,4,5]. Thus, quantifying the instability of two-phase flow not only improves the understanding of flow dynamics but also facilitates the prediction of flow pattern transitions and the optimization of operating conditions. Information entropy effectively characterizes the complexity and instability of such nonlinear dynamic systems. Although traditional single-scale entropy methods provide a macroscopic perspective [6,7,8], they fail to capture detailed multiscale characteristics. Owing to the development of multiscale entropy frameworks [9,10,11,12,13,14,15], these methods have been extensively applied in multiphase flow research.

For example, Zheng and Jin, as well as Zhou et al. [16,17,18,19,20], applied multiscale sample entropy to analyze gas–liquid two-phase flows in vertical tubes, rod bundles, inclined pipes, and horizontal pipes. They found that small-scale entropy facilitates flow pattern recognition, while large-scale trends reflect the underlying dynamic behaviors. Similarly, Gao et al. [21] adopted multivariate multiscale sample entropy to identify transient characteristics during the slug-to-churn flow transition. In studies of microchannel flows, Rafako et al. [22,23] compared multiscale and composite multiscale sample entropy and demonstrated that the complexity index from the latter is a reliable indicator of global flow complexity. To better characterize flow dynamics, various improved multiscale entropy methods have been introduced, including multiscale permutation entropy, multivariate multiscale permutation entropy, and refined composite multivariate multiscale complexity–entropy causality planes [24,25,26,27,28,29]. These methods have been successfully applied to quantifying dynamic information loss, structural evolution, and flow complexity in oil-in-water and gas–liquid two-phase flows.

Despite these advances, current multiscale entropy methods still exhibit three critical limitations. First, entropy estimation stability depends strongly on the length of the input time series; short time series often produce large fluctuations that obscure the true dynamic complexity. Second, results are highly sensitive to non-stationary abrupt changes, which disrupt the inherent regularity of the time series. Third, computational cost remains substantial due to exhaustive comparisons of subsequences or high-dimensional probability density estimation, leading to exponentially increasing processing time with time-series length.

To address these issues, this study develops a multi-resolution analysis and composite multiscale equiprobable symbolic sample entropy (MRA-CMESSE) method. MRA-CMESSE constitutes an analytical framework for characterizing flow instability in gas–liquid two-phase flow from both multistructural and multiscale perspectives. MRA lays a clear physical structural foundation by decomposing the differential pressure time series of gas–liquid two-phase flow into physically meaningful macro-scale, meso-scale, and micro-scale structures. CMESSE enables stable and reliable quantification of multiscale complexity within these structures. The parameter sensitivity and effectiveness of CMESSE have been validated using benchmark time series. On this basis, MRA-CMESSE is employed to characterize the instability characteristics of bubble, slug, and churn flows. To quantitatively describe such flow instability behaviors, two complementary metrics, namely the normalized energy (NSEnergy) and the complexity index (CI), are adopted in this work. NSEnergy reflects the scale-wise energy distribution of macro-scale, meso-scale, and micro-scale structures. CI is derived from the integral of the CMESSE curve, which quantifies the cross-scale instability of these three types of structures. The proposed MRA-CMESSE is not a trivial superposition or simple sequential combination of existing methods, but rather a physically constrained, integrated framework where CMESSE is performed on the multiscale structural components obtained by MRA. By using these two complementary metrics, this work provides a novel integrated approach for the systematic multistructure and cross-scale characterization of gas–liquid two-phase flow instability that complements and extends existing methods.

## 2. Methods

### 2.1. Multi-Resolution Analysis

Wavelet-based MRA [30,31,32,33] is an effective method for revealing nonlinear dynamic behaviors in time series. Its core principle is to iteratively decompose the approximation component $A_{j-1}$ from the (*j* − 1)-th level into a new approximation component $A_{j}$ and detail components $\left\{{D_{1},D_{2},\ldots ,D}_{j}\right\}$ by projection onto two orthogonal function sets: the scaling functions $\left\{\varphi \left(n\right)\right\}$ and the mother wavelet functions $\left\{\psi \left(n\right)\right\}$, respectively.

The corresponding basis functions are defined as:

(1) $$\varphi_{j,k}\left(n\right)=2^{j/2}\varphi \left(2^{j}n-k\right)$$

(2) $$\psi_{j,k}\left(n\right)=2^{j/2}\psi \left(2^{j}n-k\right)$$

where *j* is the current level index 1≤j≤J, *J* is the total number of decomposition levels, and $k=1,\ldots ,N/2^{j}$ is the translation parameter. The normalization factor $2^{j/2}$ ensures the orthogonality of the basis functions, which is fundamental to satisfying the energy conservation law in MRA.

For a discrete time series $x\left(n\right)$ (where n=1,2,…,N), J-level MRA decomposes $x\left(n\right)$ into a final approximation component $A_{J}$ and detail components $\left\{{D_{1},D_{2},\ldots ,D}_{J}\right\}$. For consistency, the length of components at level j is defined as $L_{j}=N/2^{j}$. The iterative process of level-*J* MRA decomposition proceeds as follows:

(a) At the first *j* = 1, $x\left(n\right)$ is projected onto the level-1 basis functions $\varphi_{1,k}\left(n\right)$ and $\psi_{1,k}\left(n\right)$, producing $A_{1}\left(k\right)$ and $D_{1}\left(k\right)$, each of length $L_{1}=N/2$:

(3) $$A_{1}\left(k\right)=\langle x(n),\varphi_{1,k}\left(n\right)\rangle =\sum_{n=1}^{L_{1}}x\left(n\right),\varphi_{1,k}\left(n\right),\;k=1,2,\ldots ,L_{1}$$

(4) $$D_{1}\left(k\right)=\langle x(n),\psi_{1,k}\left(n\right)\rangle =\sum_{n=1}^{L_{1}}x\left(n\right),\psi_{1,k}\left(n\right),\;k=1,2,\ldots ,L_{1}$$

(b) For intermediate levels 2 ≤ *j* ≤ *J* − 1, only the approximation component $A_{j-1}\left(n\right)$ with length $L_{j-1}=N/2^{j-1}$ from the preceding level is further decomposed, while all earlier detail components are preserved. Projecting $A_{j-1}\left(n\right)$ onto the *j*-th level basis functions gives:

(5) $$A_{j}\left(k\right)=\langle A_{j-1}\left(n\right),\varphi_{j,k}\left(n\right)\rangle =\sum_{n=1}^{L_{j-1}}A_{j-1}\left(n\right),\varphi_{j,k}\left(n\right),\;k=1,2,\ldots ,L_{j-1}$$

(6) $$D_{j}\left(k\right)=\langle A_{j-1}\left(n\right),\psi_{j,k}\left(n\right)\rangle =\sum_{n=1}^{L_{j-1}}A_{j-1}\left(n\right),\psi_{j,k}\left(n\right),\;k=1,2,\ldots ,L_{j-1}$$

(c) At the final *j* = *J*, the iteration concludes by projecting the (*J* − 1)-th level approximation component $A_{J-1}\left(n\right)$(with length $L_{J-1}=2$ onto J-th level basis functions:

(7) $$A_{J}\left(k\right)=\langle A_{J-1}\left(n\right),\varphi_{J,1}\left(n\right)\rangle =\sum_{n=1}^{L_{J-1}}A_{J-1}\left(n\right),\varphi_{J,1}\left(n\right),k=1,2,\ldots ,L_{J}$$

(8) $$D_{J}\left(k\right)=\langle A_{J-1}\left(n\right),\psi_{J,1}\left(n\right)\rangle =\sum_{n=1}^{L_{J-1}}A_{J-1}\left(n\right),\psi_{J,1}\left(n\right),k=1,2,\ldots ,L_{J}$$

After *J* levels of decomposition, $x\left(n\right)$ can be exactly reconstructed as the sum of all components:

(9) $$x\left(n\right)=A_{J}\left(n\right)+D_{J}\left(n\right)+D_{J-1}\left(n\right)+,\ldots ,D_{1}\left(n\right)$$

The normalized energy distribution across decomposition levels provides an intuitive measure of energy proportion at each level. By energy conservation, the total energy E of $x\left(n\right)$ equals the sum of energies of all components:

(10) $$E=E_{J}^{A}+E_{J}^{D}+E_{J-1}^{D}+,\ldots ,E_{1}^{D}=E_{J}^{A}+\sum_{j=1}^{J}E_{j}^{D}$$

where $E_{J}^{A}=\sum_{n=1}^{N_{J}}{\left|A_{J}\left(n\right)\right|}^{2},\;E_{j}^{D}=\sum_{n=1}^{N_{J}}{\left|D_{j}\left(n\right)\right|}^{2}$. The corresponding normalized energies are defined as:

(11) $$\bar{E_{J}^{A}}=E_{J}^{A}/E$$

(12) $$\bar{E_{j}^{D}}=E_{j}^{D}/E$$

In this study, the Daubechies 4 (db4) wavelet is selected as the mother wavelet, which features compact support and balanced regularity, making it well-suited for analyzing gas–liquid two-phase flow differential pressure time series [34,35,36,37]. These traits enable precise capture of transient features like Taylor bubble-induced pressure spikes while minimizing energy leakage. This is critical for preserving flow pattern-specific details such as bubble coalescence and liquid slug periodicity. Particularly, Elperin and Klochko [36] performed sensitivity comparisons of multiple wavelet bases specifically for gas–liquid two-phase flow differential pressure time series via the sparsity criterion, demonstrating the superior robustness of db4 compared to Haar and sym series wavelet bases. Similarly, Nguyen et al. [37] also validated the robustness of the db4 wavelet for similar void fraction time series. To quantitatively characterize the complexity of multiscale structural components $\left\{{{A_{J},D}_{J},D_{2},\ldots ,D}_{1}\right\}$, across scales, these components are further analyzed using CMESSE, thereby linking the multistructural decomposition obtained from MRA to a multiscale complexity representation.

### 2.2. Composite Multiscale Equiprobable Symbolic Sample Entropy

As the foundational precursor to CMESSE, equiprobable symbolic sample entropy (ESSE) quantifies time series complexity by combining equiprobable symbolization with sample entropy. This approach mitigates the bias of the original data distribution and lowers computational cost, and is computed as follows:

(a) For a time series x(*n*) of length *N*, sort all elements in ascending order to form a magnitude-ranked sequence.
(b) Given the symbol number *q*, determine *q* − 1 equiprobable thresholds $t_{1},t_{2},\ldots ,t_{q-1}$ such that each interval $\left(t_{i-1},t_{i}\right]$ contains an equal number of data points from x(*n*).
(c) Using these thresholds, map *x*(*n*) to a symbolic series $S\left(n\right)=\left\{s_{1},s_{2},\ldots ,s_{N}\right\}$, where each symbol corresponds to the interval in which the data point falls.

(13) $$s_{i}=\left\{\begin{array}{c} 0\;\;\;\;\;\;\;\;x_{i}\leq t_{1}\\ 1\;\;\;\;\;\;\;\;t_{1}<x_{i}\leq t_{2}\\ \vdots \;\;\;\;\;\;\;\;\;\;\;\;\;\;\;\;\vdots \;\;\;\;\;\;\;\;\;\;\;\;\;\;\;\;\;\;\;\;\;\;\;\;\;\;\;\;\;\;,1\leq i\leq N\\ q-2\;\;t_{q-2}<x_{i}\leq t_{q-1}\\ q-1\;\;\;t_{q-1}<x_{i}\; \end{array}\right.$$

(d) Given an embedding dimension *m*, reconstruct the symbolic series *S*(n) into m-dimensional vectors $V^{m}=\left\{v_{1},v_{2},\ldots ,v_{N-m+1}\right\}$, where $v_{i}=\left\{s_{i},s_{2},\ldots ,s_{i+m-1}\right\},i=1,2,\ldots ,N-m+1$. For each $v_{i}$, count the number of vectors $v_{j}(j\neq i)$ that are identical (i.e., all corresponding symbolic elements match), denoted as $n_{i}^{m}$. The average matching probability for *m* is:

(14) $$P^{m}=\frac{1}{N-m+1}\sum_{i=1}^{N-m+1}\frac{n_{i}^{m}}{N-m+1}$$

(e) Increase *m* to *m* + 1 and repeat the above procedure to obtain $P^{m+1}$. The ESSE value is then defined as:

(15) $$ESSE\left(q,m,N\right)=-\log\left[\frac{P^{m+1}}{P^{m}}\right]$$

Unlike traditional sample entropy, which uses a distance threshold to measure similarity, ESSE computes the exact matching probability between symbolic vectors. After equiprobable symbolization, entropy stability is governed by the equiprobable partitioning, which depends solely on *q* and *N*. For a finite *q*, the symbolic time series is robust to extreme values and effectively suppresses interference from non-stationary fluctuations.

To address the single-scale limitation of ESSE and improve entropy stability at larger scale factor τ, we integrate composite coarse-graining with ESSE to develop CMESSE. This method averages ESSE values across multiple composite coarse-grained sequences at each scale factor *τ*, thereby mitigating the effects of data distribution and non-stationary abrupt changes. The computation proceeds as follows:

(a) For a time series $x(n)=\left\{x_{1},x_{2},\ldots ,x_{N}\right\}$ and a scale factor *τ*, generate *τ* distinct coarse-grained sequences:

(16) $$y_{k}^{\tau }\left(j\right)=\frac{1}{\tau }\sum_{i=\left(j-1\right)\tau +k}^{j\tau +k-1}x_{i},\;\;\;\;1\leq j\leq \left\lfloor \frac{N}{\tau }\right\rfloor ,\;1\leq k\leq \tau ,j\tau +k\leq N$$

(b) For each $y_{k}^{\tau }\left(j\right)$ at scale *τ*, apply the same symbolization parameters *q* and *m* as in ESSE to obtain a symbolic series $s_{k}^{\tau }\left(j\right)$. Compute the counts of pairwise identical symbolic vectors in *m*- and (*m* + 1)-dimensional spaces, denoted respectively as $n_{k,\tau }^{m}$ and $n_{k,\tau }^{m+1}$.
(c) The CMESSE value at scale *τ* is the average ESSE over all *τ* coarse-grained sequences:

(17) $$CMESSE\left(q,m,N,\tau \right)=\frac{1}{\tau }\sum_{k=1}^{\tau }\log\left(n_{k,\tau }^{m+1}/n_{k,\tau }^{m}\right)$$

**Figure 1:**
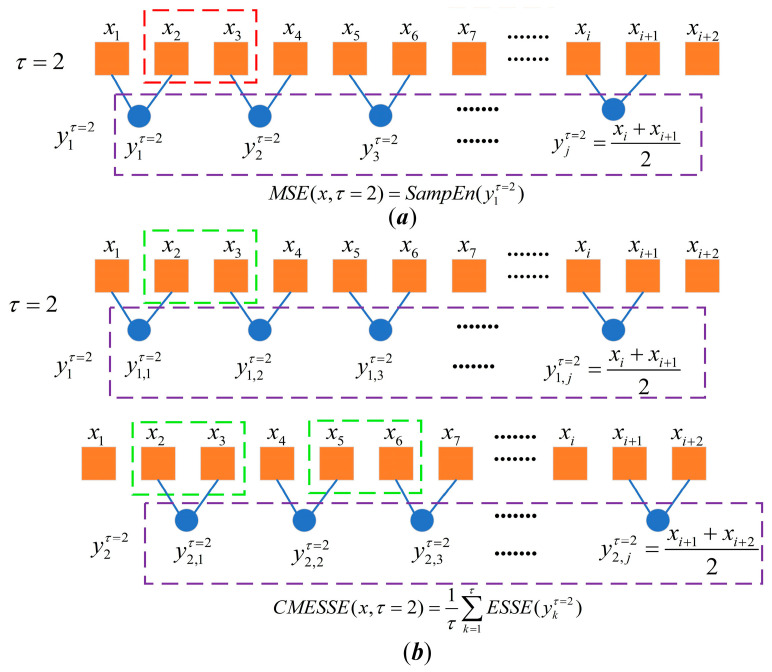
Classical coarse-graining and time-shift multiscale process. (**a**) Classical coarse-graining process; (**b**) Composite multiscale process.

Figure 1 illustrates the difference between the classical coarse-graining of MSE and the composite coarse-graining adopted in CMESSE at scale factor τ=2. As shown in Figure 1a, traditional coarse-graining only considers adjacent data pairs (e.g., $x_{1}-x_{2}$, $x_{3}-x_{4}$), neglecting correlations between other pairs (e.g., $x_{2}-x_{3}$, $x_{4}-x_{5}$), which leads to dynamic information loss. In contrast, the composite coarse-graining depicted in Figure 1b generates τ time-shifted sequences at the same scale, thereby incorporating all local data correlations of the original series and resolving the partial information loss inherent in conventional coarse-graining.

Building on the core characteristics of MRA and CMESSE described above, the two algorithms together form an integrated analytical framework that combines multistructural decomposition with multiscale complexity quantification. MRA is dedicated to decomposing nonlinear time series into multiscale structural components, while CMESSE performs multiscale complexity quantification. This synergy establishes a complete analytical chain, enabling cross-scale analysis of nonlinear dynamic behaviors from both multistructural and multiscale perspectives. The source code for the MRA-CMESSE methods presented in this paper will be publicly released upon acceptance of this work, available in a Figshare repository (https://doi.org/10.6084/m9.figshare.31869172) to support research reproducibility.

## 3. Results

### 3.1. Parameter Sensitivity Analysis

**Figure 2:**
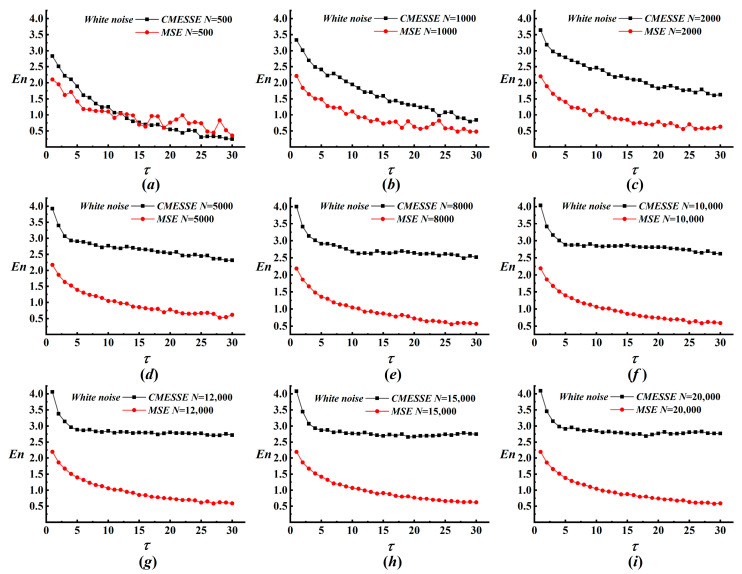
The results of CMESSE and MSE analyses for white noise with various data lengths. (**a**) *N* = 500; (**b**) *N* = 1000; (**c**) *N* = 2000; (**d**) *N* = 5000; (**e**) *N* = 8000; (**f**) *N* = 10,000; (**g**) *N* = 12,000; (**h**) *N* = 15,000; (**i**) *N* = 20,000.

**Figure 3:**
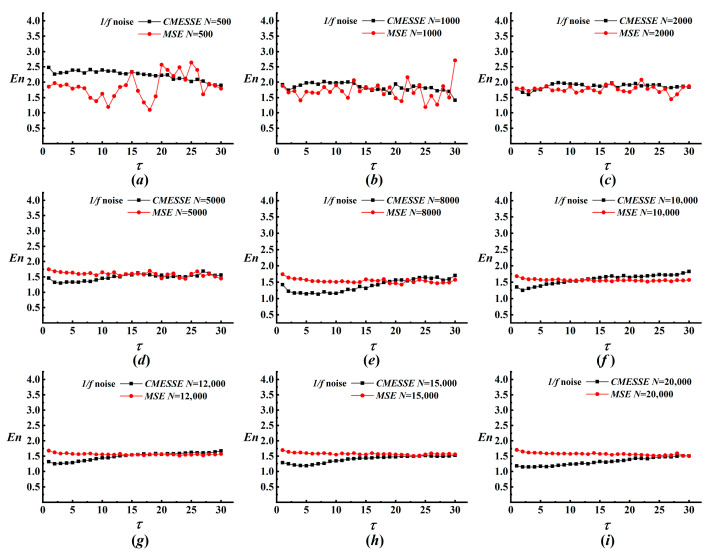
The results of CMESSE and MSE analyses for 1/f noise with various data lengths. (**a**) *N* = 500; (**b**) *N* = 1000; (**c**) *N* = 2000; (**d**) *N* = 5000; (**e**) *N* = 8000; (**f**) *N* = 10,000; (**g**) *N* = 12,000; (**h**) *N* = 15,000; (**i**) *N* = 20,000.

This section examines the parameter sensitivity of the proposed MRA-CMESSE framework. Key parameters of MRA, including the decomposition levels *J* and wavelet basis, are fixed at *J* = 8 and the db4 wavelet, based on previous studies. Thus, the performance of the overall integrated framework is primarily governed by the parameters of CMESSE. The data length *N* and the symbol number *q* are the two critical parameters in CMESSE. We first perform a sensitivity analysis of data length *N* by evaluating its influence on the stability of entropy estimation for both CMESSE and MSE over a range from 500 to 20,000 data points. The corresponding results for white noise under various data lengths are shown in Figure 2. For white noise, MSE entropy exhibits pronounced oscillations at medium-to-high scales when *N* < 5000, indicating statistical instability under limited data. In stark contrast, CMESSE yields smooth, monotonically decreasing profiles even at *N* = 500, demonstrating superior robustness to short datasets. As *N* exceeds 8000, both methods stabilize, and a fundamental divergence emerges, as detailed in Figure 2e–i. MSE entropy continues to decline monotonically across all scales. In contrast, the decay rate of CMESSE entropy gradually attenuates, converging to a nearly constant value at high scales for *N* ≥ 12,000. This finding underscores that CMESSE converges more rapidly to the theoretical asymptotic behavior of unstructured random processes. For 1/f noise, the advantage of CMESSE in small-*N* regimes is even more striking, as shown in Figure 3. MSE entropy displays large-amplitude fluctuations for *N* < 2000, whereas CMESSE trajectories remain remarkably stable. When *N* exceeds 5000, CMESSE reveals a consistent, gradual increase in entropy with scale. This phenomenon is a hallmark of long-range correlations that the inherent variability of MSE partially obscures. CMESSE substantially reduces the minimum data length required for reliable entropy estimation, achieving stable results with *N* an order of magnitude smaller than MSE. Its entropy estimates converge more rapidly and predictably with increasing *N*.

**Figure 4:**
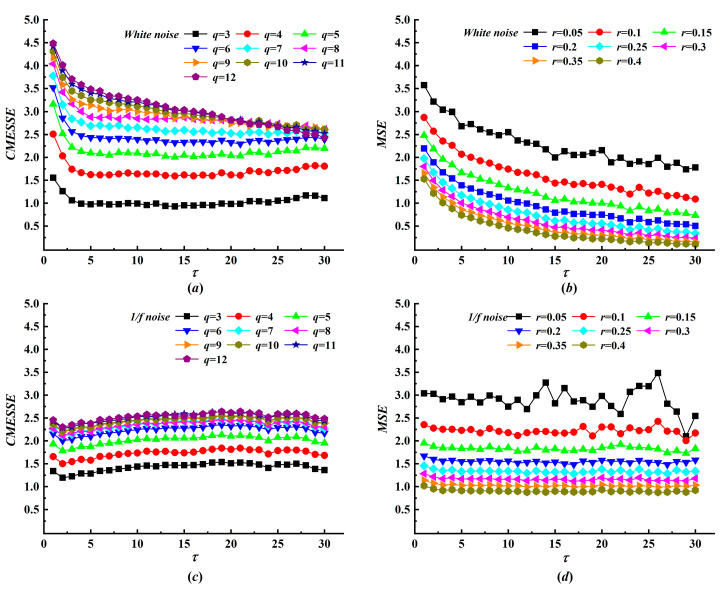
The CMESSE and MSE curves of white noise and 1/*f* noise with various symbol numbers and tolerances. (**a**) The CMESSE curves of white noise; (**b**) the MSE curves of white noise; (**c**) the CMESSE curves of 1/*f* noise; and (**d**) the MSE curves of 1/*f* noise.

Following the analysis of data length *N*, we next investigate the sensitivity of CMESSE to the symbol number *q*. In CMESSE, the symbol number *q* functions analogously to the tolerance *r* in MSE, governing the granularity of the analysis. To assess this sensitivity, we computed CMESSE entropy for white and 1/f noise across a range of *q* from 3 to 12, with fixed *N* = 10,000 and *m* = 2. As illustrated in Figure 4, CMESSE entropy for both noise types increases monotonically and smoothly with *q* at any given scale, and the curves nearly converge when *q* ≥ 8. Critically, the shape of the entropy-versus-scale curve, defined as a functional trend, remains nearly invariant to *q*. This stands in sharp contrast to MSE, where increasing *r* not only reduces entropy magnitudes but can also distort scale-dependent trends, potentially confounding physiological interpretations. The parameter *q* in CMESSE acts as a linear scaling factor rather than a distorting threshold. While a consistent *q* is required for comparing absolute entropy values, the strong invariance of the entropy profile across scales makes CMESSE far less susceptible to the arbitrary selection of this critical parameter than MSE is to *r*.

From the above sensitivity analysis, we determine *N* = 10,000, *q* = 8, *m* = 2, τ = 30 as the parameters for CMESSE in the subsequent work. The selection of *N* = 10,000 provides a reasonable trade-off between estimation stability and computational efficiency. Meanwhile, *q* = 8 generates consistent and well-behaved CMESSE profiles, avoiding issues such as insufficient granularity or information redundancy. Both parameter choices are validated by the results on white noise and 1/*f* noise. The embedding dimension *m* = 2, and *τ* = 30 are determined according to existing literature [9,10]. This parameter configuration ensures stable, consistent, and reproducible complexity evaluation.

### 3.2. Effectiveness Validation

To assess the effectiveness of CMESSE, four evaluation metrics are adopted: the coefficient of variation (CV), the entropy trend similarity index (ETSI), the entropy amplitude dissimilarity index (EADI), and the entropy robust consistency index (ERCI). A comparative analysis is conducted among CMESSE, CMSE, and MSE. We employ eight representative time series: white noise, 1/*f* noise, 1/*f*^2^ noise, a sinusoidal series sin(θ), sin(θ) with white noise, and the *Lorenz*, *Rossler*, and *Duffing* time series. The generation conditions for the chaotic series are detailed below.

*Lorenz* series is governed by:

(18) $$\left\{\begin{array}{c} \frac{dx}{dt}=\sigma (y-x)\\ \frac{dy}{dt}=x(\rho -z)-y\\ \frac{dz}{dt}=xy-\eta z \end{array}\right.$$

with parameters σ=16, ρ=45.92, η=4, and initial conditions *x*(0) = −1, *y*(0) = 0, *z*(0) = 1.

*Rossler* series is described by:

(19) $$\left\{\begin{array}{c} \frac{dx}{dt}=-y-z\\ \frac{dy}{dt}=x-ay\\ \frac{dz}{dt}=\left(x-b\right)z+c \end{array}\right.$$

where a=0.15,b=10, c=0.2, and the initial conditions are *x*(0) = −1, *y*(0) = 0, *z*(0) = 1.

*Duffing* series follows the equation:

(20) $$\ddot{x}+k\dot{x}-ax+cx^{3}=fcos(\omega t)$$

With parameters *k* = 0.05, *a* = 0.5, *c* = 1, forcing amplitude *f* = 7.5 and frequency ω = 1. Initial conditions are set as $x\left(0\right)=-1,\;\;\dot{x}(0)=0$.

All series were standardized to a length of *N* = 10,000. For consistency across methods, the following parameters were fixed at *m* = 2, *q* = 8 for CMESSE, and *r* = 0.2 for MSE and CMSE. The stability of entropy calculation across scales can be evaluated using the CV, where lower CV values correspond to more stable outputs. CMESSE exhibits enhanced stability in multiscale entropy analysis by consistently producing the lowest CV values across all tested sequences. As shown in Table 1, CMESSE achieves a CV of 0.099 for white noise, which is markedly lower than the values of 0.429 from CMSE and 0.443 from MSE. This advantage persists across other signal categories, with CMESSE recording 0.0404 for 1/*f* noise and 0.2081 for sine wave with white noise mixed signals, both substantially lower than the corresponding values from the comparative methods. For chaotic sequences, including those from the *Lorenz*, *Rossler*, and *Duffing* systems, all three approaches demonstrate similar capability in characterizing complex dynamics. Nevertheless, CMESSE retains a slight stability advantage. with CV values of 0.256, 0.1589, and 0.2868, respectively, which are comparable to or lower than those from CMSE and MSE.

Results of two-tailed paired *t*-tests on 90 groups of chaotic time series (30 independent groups for each of the *Lorenz*, *Rossler*, and *Duffing* systems) with 20% white Gaussian noise added are presented in Table 2. For reproducibility, fixed random seeds (seed = 123 + group index) were used to generate 20% white Gaussian noise for each group. The white noise intensity was set to 20% of the standard deviation of the normalized original time series (mean = 0, std = 1). The generated noise was further standardized to zero mean and unit standard deviation before addition to avoid intensity deviation. CMESSE presents lower CV values and better consistency than CMSE in the Lorenz and Rossler time series. In the Duffing time series, CMESSE shows a slightly higher CV value and relatively weaker consistency. The statistic *t*(29) describes the magnitude and direction of the difference between the two methods. A positive sign indicates that CMESSE outperforms CMSE, while a negative sign indicates the opposite trend. All *p*-values are less than 0.01, demonstrating that the differences between CMESSE and CMSE are statistically significant.

CMESSE consistently demonstrates clear superiority based on the composite performance metrics detailed in Table 3. It achieves higher trend synchronization scores across all sequence groups, with 0.9917 for noise, 0.908 for chaotic sequences, and 0.931 overall, all exceeding the results from both reference methods. For amplitude consistency, CMESSE again demonstrates better performance, with EADI values of 0.099 for noise sequences and 0.284 for all sequences, substantially lower than those obtained by CMSE and MSE. The comparable amplitude consistency results across methods for chaotic sequences reflect inherent data characteristics rather than methodological limitations. Most notably, in the comprehensive robustness assessment, CMESSE scores 0.848 for noise sequences, 0.736 for chaotic sequences, and 0.752 for all sequences, with ERCI values that remain consistently higher than competing methods. These findings confirm that CMESSE provides more consistent trend characterization and more stable amplitude estimation than existing methods, making it particularly effective for analyzing complex or heterogeneous time series data.

## 4. Discussion

### 4.1. Experimental Set-Up and Data Acquisition

**Figure 5:**
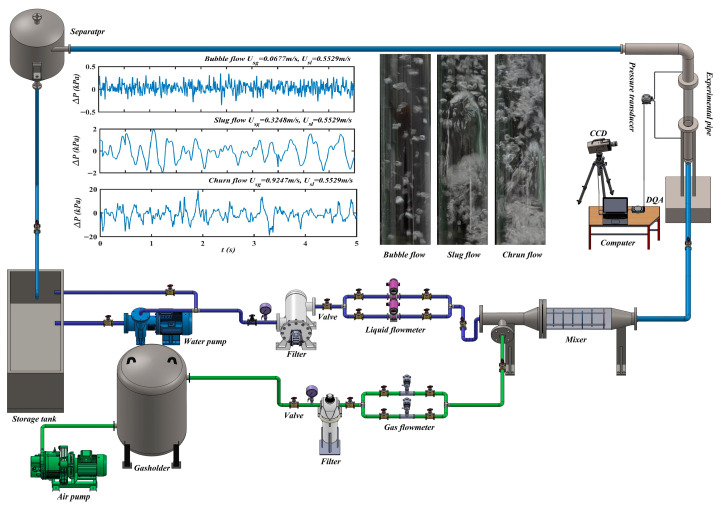
Experimental measurement system of air–liquid two-phase flow in vertical upward pipe.

The differential pressure time series acquired from the experiment are first decomposed by an eight-level MRA, then analyzed by CMESSE to calculate NSEnergy and CI. The schematic of the experimental setup for air–water two-phase flow in a vertical upward pipe is shown in Figure 5. The system consists of four main modules: a working fluid supply system, an operating condition control unit, a measurement pipe section, and a data acquisition and visualization system. The gas phase is compressed air from an air compressor, whose superficial gas velocity is measured using a gas mass flowmeter. The liquid phase is room-temperature water supplied from a storage tank, with its flow rate measured using a turbine flowmeter. The air–water two-phase mixture is fully mixed before entering the test pipe, which has an inner diameter of 40 mm and a length of 1200 mm. During each experimental run, the pipe is first filled with water at a constant liquid flow rate. Subsequently, different flow patterns are established by adjusting the gas flow control valve. Once the flow stabilizes, the differential pressure fluctuation time series and synchronized flow images are recorded simultaneously using a differential pressure transducer, a data acquisition card (DAQ), and a high-speed camera. To guarantee data reliability, the distance between pressure taps is set to 400 mm, the sampling frequency is set to 2000 Hz, and the acquisition duration is set to 5 s.

**Figure 6:**
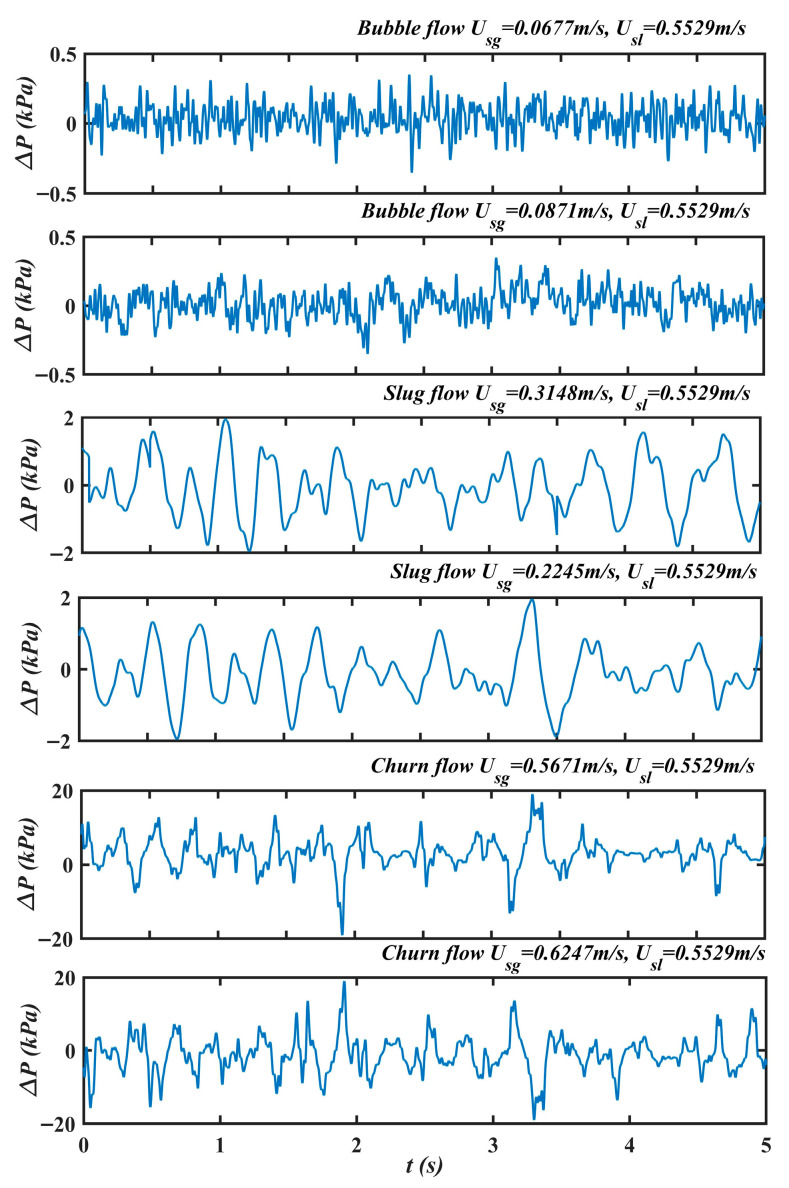
The differential pressure fluctuation time series of bubble, slug and churn flow.

Experiments are conducted over a range of gas–liquid superficial velocity ratios. The superficial gas velocity $U_{sg}$ varies from 0.01 to 2.5 m/s, while the superficial liquid velocity $U_{sl}$ is held constant at 0.5305 m/s. Three typical two-phase flow patterns including bubble, slug, and churn flow are observed during the experiments. The corresponding differential pressure fluctuation time series are displayed in Figure 6. In bubble flow, the gas fraction is relatively low and the bubbles are well separated. The continuous liquid phase strongly suppresses bubble coalescence, resulting in low-amplitude, quasi-random fluctuations at the gas–liquid interface. Consequently, the differential-pressure signal exhibits small-amplitude, aperiodic oscillations. In slug flow, a higher gas fraction leads to the merging of small bubbles into large Taylor bubbles that almost fully occupy the pipe cross section. The alternating passage of these Taylor bubbles and liquid slugs, together with the downward drainage of the liquid film along the pipe wall, generates larger-amplitude interfacial motions. The corresponding differential-pressure time series displays evident pseudo-periodic fluctuations. In churn flow, a further increase in gas fraction destabilizes Taylor bubbles and breaks them into irregular structures of varying sizes. This enhances turbulent fluctuations at the gas–liquid interface, leading to highly irregular, large-amplitude interfacial oscillations. As a result, the differential-pressure signal shows strong, chaotic fluctuations.

### 4.2. Flow Instability Analysis of Gas–Liquid Two-Phase Flow Based on MRA and CMESSE

**Figure 7:**
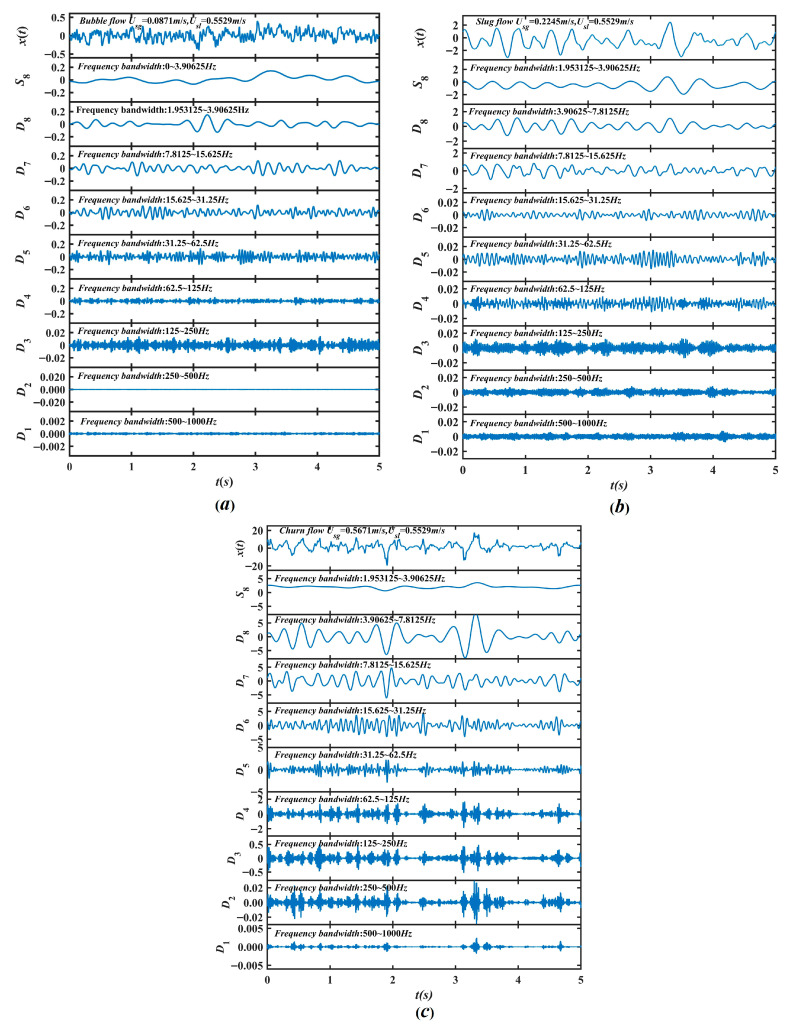
The results of MRA for bubble, slug and churn flow. (**a**) bubble flow (U_sg_ = 0.0871 m/s, U_sl_ = 0.5529 m/s); (**b**) slug flow (U_sg_ = 0.2245 m/s, U_sl_ = 0.5529 m/s); (**c**) churn flow (U_sg_ = 0.5671 m/s, U_sl_ = 0.5529 m/s).

Flow instability in gas–liquid two-phase flow is intrinsically linked to the nonlinear dynamics of its multiscale flow structures. MRA provides an effective approach to characterize these dynamics by decomposing signals into macro-, meso-, and micro-scale components [31,32,33]. In this study, an 8-level MRA is performed to decompose each differential pressure time series under bubble, slug, and churn flow conditions into eight detail components *D*_1_–*D*_8_ and one approximation component $A_{8}$, as shown in Figure 7. The frequency range of each detail component is [*f_s_*/(2^(*j* + 1)^), *f_s_*/(2*^j^*)], where *f_s_* = 2000 Hz denotes the sampling frequency, and the approximation component $A_{8}$ covers the frequency range [0, *f_s_*/(2^9^)] Hz.

**Figure 8:**
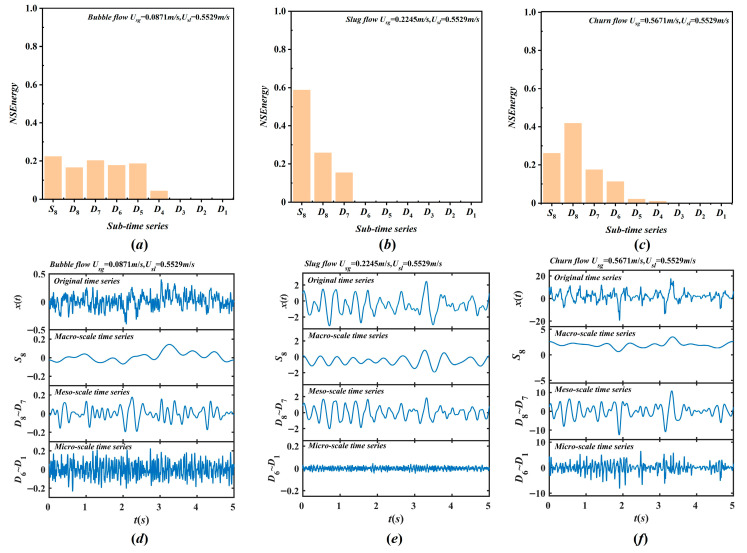
The MRA NSEnergy and multiscale structural time series in different flow patterns. (**a**) MRA NSEnergy of bubble flow; (**b**) MRA NSEnergy of slug flow; (**c**) MRA NSEnergy of churn flow; (**d**) multiscale structural time series of bubble flow; (**e**) multiscale structural time series of slug flow; (**f**) multiscale structural time series of churn flow.

Based on energy conservation, the normalized signal energy (NSEnergy) at each structural scale is calculated and presented in Figure 8a–c. Figure 8a shows that the NSEnergy in bubble flow is mainly concentrated in $A_{8}$ and D_4_–D_8_, owing to the global void motion. Energy is distributed across macro-, meso-, and micro-structures, corresponding to the low-frequency fluctuating characteristics of bubble flow. The NSEnergy of $A_{8}$ represents the macroscopic instability associated with global flow motion. D_7_–D_8_ capture the instability related to bubble agglomeration, dispersion, and local cluster structures. D_6_–D_1_ correspond to small bubble surface deformations and local liquid phase interactions. As illustrated in Figure 8b, in contrast to bubble flow, the NSEnergy of slug flow is dominantly concentrated in $A_{8}$ and D_7_–D_8_, with an extremely high proportion in $A_{8}$ and very little in D_6_–D_1_. The NSEnergy of $A_{8}$ reflects the macroscopic instability induced by the alternating motion of Taylor bubbles and liquid slugs. D_7_–D_8_ characterize the interfacial interactions between these structures. D_6_–D_1_ mainly describe micro-scale behaviors and turbulence of small bubbles and droplets within the slug flow. Figure 8c presents the NSEnergy distribution for churn flow, which shows a concentration in $A_{8}$ and D_7_–D_8_ similar to bubble flow, with a key distinction that the NSEnergy in D_7_–D_8_ is considerably higher. The NSEnergy of $A_{8}$ mainly reflects the global macroscopic structural motion, while D_7_–D_8_ represent the motion instability of localized structures, including bubble clusters and droplets. D_6_–D_1_ mainly correspond to micro-scale structural instability and turbulence intensity.

This decomposition enables the reconstruction of multiscale components from the original time series, namely macro-, meso-, and micro-scale structures. The definition of these three composite time series in Figure 8d–f is not only a mathematical result derived from an 8-level dyadic wavelet decomposition, but also supported by the intrinsic physical mechanisms of gas–liquid two-phase flow and existing literature [34,35,36,37]. The macro-scale series (A_8_, 0–3.90625 Hz) corresponds to the passage of Taylor bubbles and periodic oscillations of liquid slugs, and reflects global pressure drop fluctuations. This division is consistent with the characteristic low-frequency range (<4 Hz) reported in previous studies [34,36], and the NSEnergy proportion of A_8_ further validates its physical significance as an indicator of global flow motion. The meso-scale series (D_7_–D_8_, 3.90625–15.625 Hz) is associated with bubble coalescence, breakup, and cluster dynamics, corresponding to the collective motion of bubble clusters that bridge global and local flow structures. This interpretation is supported by Farge [35] and Nguyen et al. [37], and our NSEnergy results provide further validation. D_7_–D_8_ exhibits a concentrated energy distribution across all flow patterns, in agreement with the corresponding cluster dynamics. The micro-scale series (D_1_–D_6_, 15.625–1000 Hz) mainly corresponds to the micro-scale phenomena, including bubble deformation, interfacial oscillation, and local turbulence. These micro-scale mechanisms endow the signal with wideband spectral characteristics [35]. The continuous, flat energy distribution of D_1_–D_6_ in high frequencies also confirms this interpretation. Furthermore, high-frequency detail components are generally classified as the micro-scale in wavelet-based analyses of gas–liquid two-phase flow [36,37].

**Figure 9:**
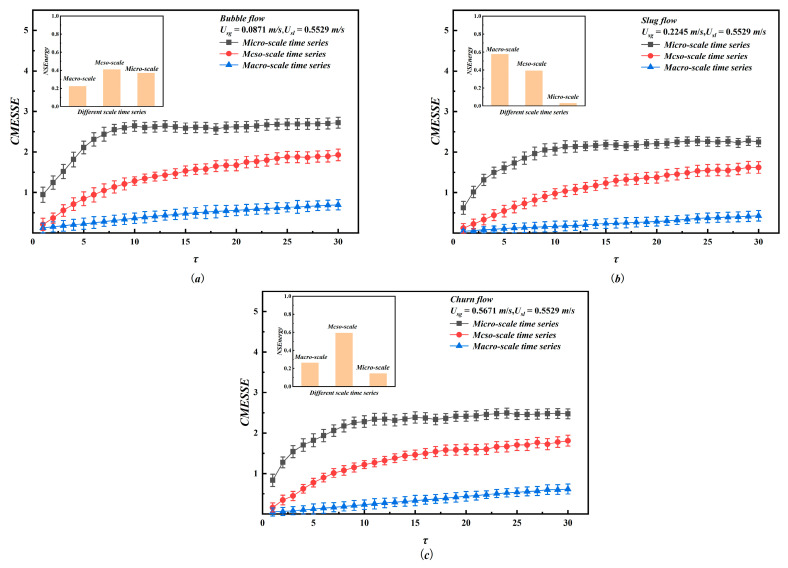
The CMESSE and NSEnergy of different scale time series in different flow patterns. (**a**) bubble flow (U_sg_ = 0.0871 m/s, U_sl_ = 0.5529 m/s); (**b**) slug flow (U_sg_ = 0.2245 m/s, U_sl_ = 0.5529 m/s); (**c**) churn flow (U_sg_ = 0.5671 m/s, U_sl_ = 0.5529 m/s).

Figure 9 further integrates NSEnergy with CMESSE. Bubble flow exhibits a broad NSEnergy distribution across all scales. The macro-scale accounts for 22.38%, the meso-scale for 40.88%, and the micro-scale for 36.74%. The low macro-scale CMESSE, remaining below 0.7, reflects the stable global transport of dispersed bubbles within the pipe. In contrast, the higher meso-scale CMESSE, ranging from 0.214 to 1.926, and the highest micro-scale CMESSE, from 0.945 to 2.722, characterize, respectively, the organized but random aggregation of bubble clusters and the intense random motion and collisions of individual bubbles. The considerable energy proportion and high complexity at the micro-scale serve as key indicators of the relatively high overall instability of bubble flow. Slug Flow shows a concentrated NSEnergy distribution. The macro-scale accounts for 57.68% of the total energy, the meso-scale for 39.31%, and the micro-scale for only 3.1%. The very macro-scale CMESSE, ranging from 0.045 to 0.427, corresponds to the orderly quasi-periodic alternating motion of Taylor bubbles and liquid slugs. The moderate meso-scale CMESSE, ranging from 0.214 to 1.926 stems from local interfacial disturbances between these structures. Although the micro-scale CMESSE spans a wide range, from 0.623 to 2.278, its negligible energy contribution indicates that slug flow has the lowest instability among the three patterns, as it is dominated by large-scale coherent motions. Churn flow presents a distinct distribution in which NSEnergy peaks at the meso-scale 59.3%, with lower contributions from the macro-scale 26.16% and the micro-scale 14.54%. The low macroscale CMESSE 0.051 to 0.618 indicates preserved large-scale coherence. However, the dominant meso-scale energy, combined with CMESSE values from 0.162 to 1.812, signals strong chaotic interactions induced by the breakup and coalescence of large bubbles. The elevated micro-scale CMESSE 0.833 to 2.494 further confirms intense small-scale turbulence. Therefore, churn flow exhibits intermediate instability, dominated by strong meso-scale dynamic behaviors.

**Figure 10:**
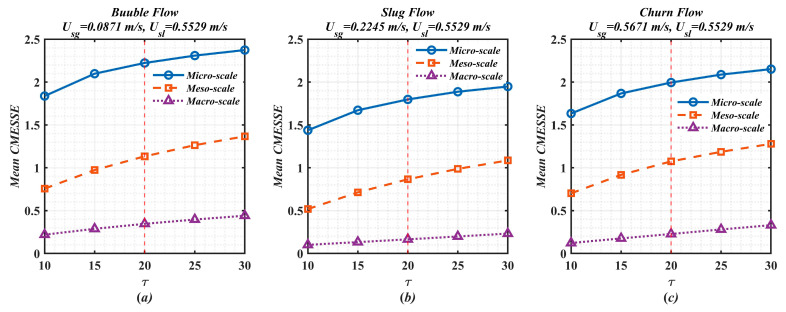
The mean CMESSE variation with τ for different flow patterns. (**a**) bubble flow; (**b**) slug flow; (**c**) churn flow.

The Complexity Index (CI) is a cumulative metric that exhibits an inherent monotonic increase with the scale factor τ, representing the total multiscale complexity of the macro-, meso-, and micro-scale structures. To analyze the sensitivity of CI to τ, we introduce the mean CMESSE, defined as CI(τ)/τ, which quantifies the average complexity per unit scale. The convergence trend of the mean CMESSE is presented in Figure 10. It can be observed that the mean CMESSE for all three flow patterns and their corresponding micro-, meso-, and macro-scale structures increases with increasing τ, while its growth rate gradually decreases. Taking bubble flow as an example, the growth increment of the mean CMESSE for the micro-scale structure decreases monotonically from 0.2607 over τ = 10–15 to 0.0646 in τ = 25–30, and analogous decreasing increments are observed for the meso- and macro-scale structures. This consistent slowing trend indicates that the mean CMESSE will not increase without bound, implying that CI does not grow unboundedly with τ.

**Figure 11:**
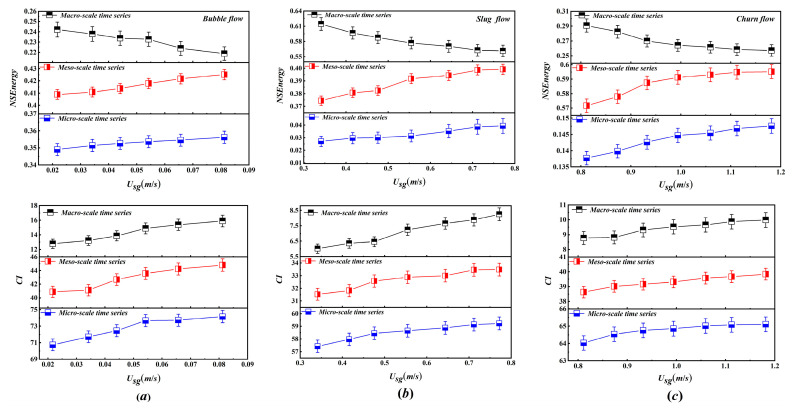
The NSEnergy and CI in macro-, meso- and micro-scales time series: Trend with increasing U_sg_ under bubble, slug and churn flow conditions. (**a**) bubble flow; (**b**) slug flow; (**c**) churn flow.

To investigate the effect of U_sg_ on the evolution of unsteadiness, the NSEnergy and complexity index (CI) are calculated for the three flow patterns at different U_sg_ values and a fixed U_sl_ = 0.5305 m/s. As U_sg_ increases, a clear transfer of NSEnergy from the macro-scale toward the meso- and micro-scales is observed for all flow patterns in Figure 11. For bubble flow, macro-scale NSEnergy decreases from 0.2421 to 0.2187, while meso- and micro-scale NSEnergy increase from 0.4088 to 0.4251 and from 0.3491 to 0.3562, respectively. In slug flow macro-scale NSEnergy declines from 0.6142 to 0.5615, meso-scale NSEnergy rises from 0.3744 to 0.3991, and micro-scale NSEnergy increases markedly from 0.0014 to 0.0394. For churn flow, macroscale NSEnergy decreases from 0.2907 to 0.2572, meso-scale NSEnergy increases from 0.5716 to 0.5952, and micro-scale NSEnergy rises from 0.1377 to 0.1476. This energy redistribution is attributed to the enhanced drag effect of the gas phase on the liquid phase, which generates more turbulence and vortex structures, thereby reducing the stability of the gas–liquid interface. The consequent destabilization across all structural scales explains why CI increases with increasing U_sg_. Since gas–liquid two-phase flow behavior is closely related to the ratio of U_sg_ to U_sl_, NSEnergy and CI are also affected by U_sl_. A higher liquid velocity would normally strengthen interfacial shear, turbulent kinetic energy, and the breakup of large-scale interfacial structures, which in turn affects the overall intensity of flow instability. From this perspective, changing Usl would primarily influence the absolute magnitudes of NSEnergy and CI, rather than the qualitative evolution trends across scales.

It is noteworthy that the quantitative response varies. The decrease in macro-scale NSEnergy is more pronounced in slug and churn flows than in bubble flow. The increase inmacro-scalee CI is greatest in slug flow, whereas the increase in macro-scale CI is most significant in bubble flow. These differences are linked to inherent flow structures. Compared to bubble flow, slug and churn flows contain more macro-structures that can be readily transformed into meso- and micro-structures under elevated turbulent energy, especially in churn flow. Furthermore, across all patterns, the CI of the meso- and micro-scale time series exhibits substantially greater increases than that those of tmacro-scaleale series. This implies that instability at smaller scales responds more readily to increases in U_sg_ and accounts for a larger portion of the overall rise in flow instability.

## 5. Conclusions

This study develops and validates an integrated MRA-CMESSE framework for characterizing gas–liquid two-phase flow instability. This framework effectively improves the limited stability of entropy estimation and the insufficient physical interpretability in multiphase flow dynamic analysis. It reveals the evolution mechanism of flow instability using normalized energy and the complexity index across macro-, meso- and micro-scale structures. Results show that bubble flow shows a broad NSEnergy distribution across macro-, meso- and micro-scales with the highest complexity index. Slug flow is dominated by macro-scale NSEnergy with the lowest complexity index. Churn flow has the largest meso-scale NSEnergy proportion with a moderate complexity index. In addition, increasing gas superficial velocity shifts energy from the macro-scale to the meso- and micro-scales. Smaller-scale structures respond more strongly to gas velocity variations.

The established framework offers an innovative methodological tool and refined physical insight for predicting and controlling flow patterns in engineering systems such as energy and chemical processing. A limitation of the present work is that only a single liquid superficial velocity is considered. Future work will explore a wider range of liquid velocities to further examine their influence on flow instability and energy evolution characteristics.

## Figures and Tables

**Table 1:** Coefficient of Variation (CV) for CMESSE, CMSE and MSE for Representative Time Series.

Serial No.	Time Sequence	CMESSE CV	CMSE CV	MSE CV
1	White noise	0.099	0.4294	0.443
2	1f noise	0.0404	0.0733	0.0724
3	1f2 noise	0.3679	0.4069	0.4077
4	Sine	0.4885	0.6186	0.6203
5	Sine + White Noise	0.2081	0.5416	0.5447
6	*Lorenz*	0.256	0.29	0.2908
7	*Rossler*	0.1589	0.2167	0.2188
8	*Duffing*	0.2868	0.2921	0.2866

**Table 2:** Paired *t*-test results of CMESSE and CMSE for chaotic time series with 20% white noise.

Chaotic Time Series	CMESSE CV (Mean ± SD)	CMSE CV (Mean ± SD)	*t*(29)	*p*
Lorenz	0.224 ± 0.003	0.239 ± 0.002	19.4222	<0.01
Rossler	0.114 ± 0.003	0.165 ± 0.004	60.8353	<0.01
Duffing	0.268 ± 0.010	0.248 ± 0.005	−13.9347	<0.01

**Table 3:** Comparison Table of ETSI, EADI and ERCI metrics for CMESE, CMSE and MSE.

Statistical Index	Sequence Group	CMESSE	CMSE	MSE
Entropy Trend Similarity Index (ETSI)	Noise	0.9917	0.9126	0.9098
	Chaotic	0.9083	0.8067	0.8064
	All Sequences	0.9313	0.8847	0.8827
Entropy Amplitude Dissimilarity Index (EADI)	Noise	0.0992	2.2646	2.2909
	Chaotic	1.597	1.8463	1.827
	All Sequences	0.2842	0.878	0.8992
Entropy Robust Consistency Index (ERCI)	Noise	0.8483	0.7206	0.6957
	Chaotic	0.7361	0.637	0.6373
	All Sequences	0.7521	0.6512	0.6488

## Data Availability

The raw data supporting the conclusions of this article will be made available by the authors on request.
